# Separation of GVL from GVHD -location, location, location

**DOI:** 10.3389/fimmu.2023.1296663

**Published:** 2023-12-05

**Authors:** Takanori Teshima, Daigo Hashimoto

**Affiliations:** Department of Hematology, Hokkaido University Faculty of Medicine, Sapporo, Japan

**Keywords:** graft-versus-leukemia, graft-versus-host disease, intestine, tumor immune environment, tissue tolerance

## Abstract

Allogeneic hematopoietic cell transplantation (HCT) is a curative therapy for various hematologic malignancies. However, alloimmune response is a double-edged sword that mediates both beneficial graft-versus-leukemia (GVL) effects and harmful graft-versus-host disease (GVHD). Separation of GVL effects from GVHD has been a topic of intense research to improve transplant outcomes, but reliable clinical strategies have not yet been established. Target tissues of acute GVHD are the skin, liver, and intestine, while leukemic stem cells reside in the bone marrow. Tissue specific effector T-cell migration is determined by a combination of inflammatory and chemotactic signals that interact with specific receptors on T cells. Specific inhibition of donor T cell migration to GVHD target tissues while preserving migration to the bone marrow may represent a novel strategy to separate GVL from GVHD. Furthermore, tissue specific GVHD therapy, promoting tissue tolerance, and targeting of the tumor immune microenvironment may also help to separate GVHD and GVL.

## Introduction

Allogeneic hematopoietic cell transplantation (HCT) is a curative treatment for leukemia and other various hematologic malignancies. Its antileukemic effect is mediated by donor immune cells and refers as graft-versus-leukemia (GVL) effects. This phenomenon was recognized already in 1956; Barnes et al. reported that leukemia-bearing mice receiving allogeneic cells eventually died of GVHD without evidence of leukemia (1). Weiden et al. documented its clinical effects on preventing relapse in 1979 (2). Since then, the goal for HCT remains the enhancement of GVL effect while limiting GVHD. However, GVL activity is clearly associated with GVHD; patients with acute GVHD or chronic GVHD have a significantly lower risk of relapse after HCT compared to those without GVHD (2, 3).

**Figure 1:**
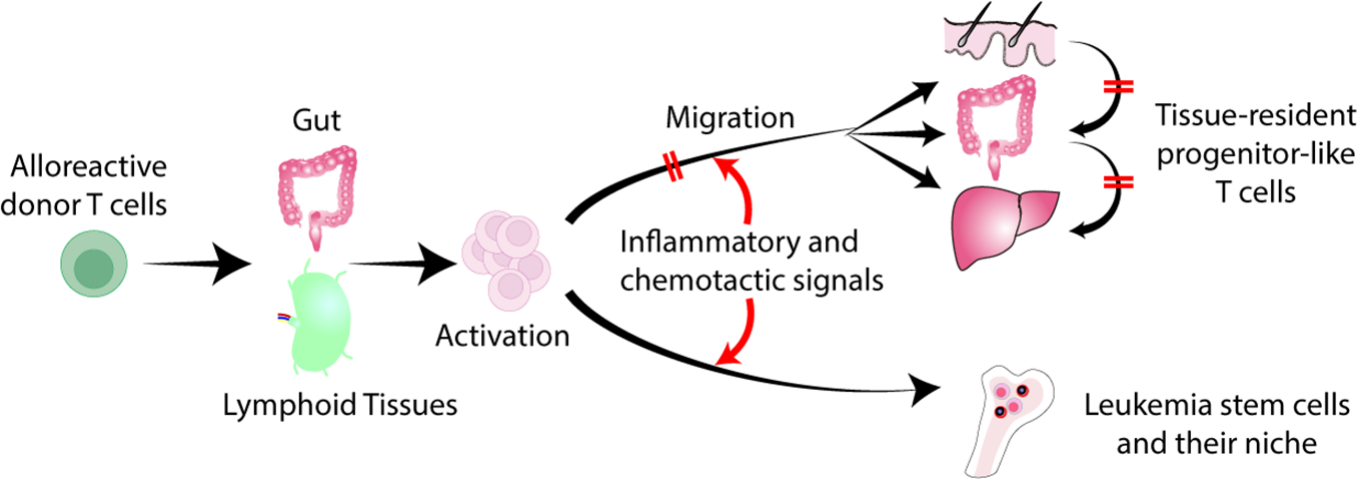
Separation of GVL from GVHD by regulating T cell trafficking to tissues. Donor derived alloreactive T cells are activated in the gut and secondary lymphoid organs, and migrate into the skin, liver, and gut. Tissue-specific effector T cell migration is determined by a combination of inflammatory and chemotactic signals that interact with specific receptors on T cells. Inhibition of donor T cell migration to GVHD target tissues without impeding T cell trafficking to the bone marrow represents a novel strategy to separate GVL from GVHD. Late-phase GVHD is maintained within affected tissues locally by tissue-resident TCF1^+^ pTex-like cells. Local inhibition of these cells may not impede GVL.

Separation of GVL effects from GVHD has been a topic of intense research to improve transplant outcomes. Over the past several decades, clinical attempts to identify and separate specific immune effector mechanisms that can dissect GVHD and GVL have been unsuccessful. Current therapies for GVHD target T cells and cytokines, often antagonizing GVL effects. Acute GVHD targets specific tissues, such as the skin, gut, and liver. Recruitment of donor T cell into these tissues plays a major role in GVHD (4). On the other hand, bone marrow niche is commonly observed in various hematologic malignancies (5). Leukemia stem cells (LSCs) are resistant to chemotherapy, making them the drivers of leukemia relapse, and they reside in the bone marrow niche (6, 7). Tissue-specific effector T cell emigration is determined by a complex milieu of inflammatory and chemotactic signals that interact with specific receptors on T cell (8). Modulation of donor T cell migration may pave a new avenue to separate GVHD and GVL (**Figure 1**).

Similar to T cells, natural killer (NK) cells have potent anti-leukemia effector capacity, but unlike T cells, NK cells have less ability to mediate GVHD. Because of their lack of HLA-restricted specificity, allogeneic NK cells can be administered across HLA barriers without GVHD (9). Recent studies have shown that donor NK cell infusion after haploidentical bone marrow transplantation using posttransplant cyclophosphamide (PTCy-haplo) reduced relapse compared to historical controls, with an excellent safety profile (10). Donor selection according to killer immunoglobulin-like receptor (KIR) alloreactivity is associated with superior survival in PTCy-haplo (11). Maximizing donor NK alloreactivity thus holds the exciting possibility to induce GVL effect without engendering GVHD. In this review, we will discuss current attempts to separate GVHD and GVL as well as novel concepts of GVHD and GVL separation by considering the location of GVHD and GVL.

## Donor T-cell and antigen-presenting cell interactions mediating GVHD and GVL

The risk of leukemia relapse was significantly higher in patients who did not develop GVHD or who received a T cell-depleted graft or a graft from patient’s identical twin (12, 13). Thus, donor T cells play a major role in mediating GVL effects. Shlomchik et al. demonstrated that naïve T cells rather than memory T cells played the major role in inducing GVHD in mice (14, 15). Clinical trial data of naïve T cell-depleted HCT demonstrated low incidences of severe acute GVHD and chronic GVHD without apparent excess risks of leukemia relapse (16, 17), but effect of naïve T cell depletion on leukemia relapse remains to be evaluated in larger studies. Unlike the memory T cells developed in donor, donor memory cells developed in the recipients after allo-SCT are involved in GVHD. It has been shown that donor stem cell-like memory cells (Tscm) persist in the recipient and maintain alloreactivity against host alloantigens in mice. Adoptive transfer of Tscm into the secondary recipients induced GVHD (18). Tscm population has been identified in human (19). A recent study showed that administration of PTCy spared Tscm that can improve GVL (20).

Donor T cells mediating both GVHD and GVL are activated primarily by recipient antigen presenting cells (APCs) in mice (21–23). Reconstituting donor hematopoietic APCs cross-present host antigens to invoke the full spectrum of GVHD and GVL (22, 24–26). Thus, separation of GVL from GVHD could not be easily achieved by the modulation of donor T cell and APC interactions.

After allogeneic HCT, host-derived alloantigen persists lifelong, which could induce T cell exhaustion. A series of experimental studies showed that T cell exhaustion is one of the chief mechanisms of tolerance induction without chronic GVHD after allogeneic HCT (27–29). T cell exhaustion is a multistep process, involving precursors of exhausted T cells (Tpex) with stem cell-like properties, transitory exhausted T cells (transitory-Tex) with potent effector-like functions and terminally differentiated exhausted T cells (terminal-Tex) with severely impaired functions (30–33). Calcineurin inhibitors inhibit T-cell exhaustion by inhibiting expression of a master regulator of T cell exhaustion, TOX (34). In experimental HCT, GVHD prophylaxis with calcineurin inhibitors suppresses differentiation of transitory-Tex to terminal-Tex, resulting in persistent alloreactivity and induction of chronic GVHD (35). T cell dysfunction of terminal-Tex is irreversible and non-responsive to immune checkpoint inhibitors (ICI), while ICI enhance proliferation and effector functions of Tpex and transitory-Tex (36). Therefore, calcineurin-induced transitory-Tex could be a promising therapeutic target to restore GVL effects by ICI but with a risk of GVHD exacerbation (35).

## Target antigens mediating GVHD and GVL

GVHD and GVL are mediated by donor T cells recognizing non-self-antigens expressed on host- and donor-derived APCs. In HLA-identical HCT, GVHD is induced by minor histocompatibility antigens (miHA), which are HLA-bound peptides that differ between the donor and recipient due to genetic polymorphisms (37). Although it remains unclear how many immunodominant miHA could evoke significant GVHD and GVL in humans, most miHA are ubiquitously expressed including epithelial tissues, thereby potentially inducing GVHD.

miHA with expression limited to hematopoietic cells represent attractive candidate targets for selective induction of GVL effects without causing GVHD in patients with hematologic malignancies. For example, adoptive transfer of H7^a^ (B6^dom1^) specific T cells eradicates H7^a^-expressing leukemia efficiently in H7^a^-deficient mice without GVHD (38). Transfer of H60-specific CD8^+^ memory T cells eradiates chronic myeloid leukemia cells (39). Several studies have suggested that GVL activity is greater against hematopoietic restricted miHA by eliciting less exhaustion and activation-induced cell death of alloreactive T cells than that against ubiquitously expressed miHA (28, 39). In humans, cytotoxic T lymphocytes (CTLs) specific for male tissue specific miHA H-Y induce skin injury when co-cultured with male skin biopsy specimen, while hematopoietic system-specific miHA HA-1 and HA-2 induce little tissue injury (40). However, an inflammatory environment can render nonhematopoietic cells susceptible to T cell recognition to induce GVHD (41). It is also challenging to identify candidate miHA that can be widely applied to heterogenous patient-donor combinations. Well characterized hematopoietic miHA in humans include HA-1, HA-2, ACC-1, ACC-2, and LRH1 (40, 42–45). It remains to be elucidated whether mismatch of these miHA could reduce leukemia relapse without inducing GVHD in the presence of the other multiple miHA mismatches between the donor and recipient. Immunotherapy using specific miHA-directed T cells generated by genetic modification or vaccination is promising strategy but remains to be evaluated in prospective comparative studies.

## Donor T cell migration

Acute GVHD is organ-specific and principally affecting the skin, liver, and gut. Development of GVHD requires donor T cells to migrate into these tissues (4). Several tissue-specific T cell homing receptors have been identified. The α4β7 integrin is critical for T cell homing to the gastrointestinal tract and gut-associated lymphoid tissues (46). T cell migration to the skin is directed by cutaneous lymphocyte antigen, a specialized form of P-selectin glycoprotein ligand-1 (47). Regulation of effector T cell migration into target tissues occurs in a complex milieu of chemotactic signals where several receptors may be triggered simultaneously or successively (48). Inflammatory chemokines expressed in inflamed tissues upon stimulation by proinflammatory cytokines are specialized for the recruitment of donor T cells and other effector cells (8, 49). Chemokine receptors are differentially expressed on subsets of activated/effector T cells. Upon stimulation, T cells rapidly switch chemokine receptor expression and acquire new migratory capacity (50, 51). Requirement of donor T cell homing to specific tissues has profound clinical implications to modulate GVHD and GVL.

Pharmaceutical agents used for prophylaxis and treatment of GVHD such as calcineurin inhibitors, antimetabolites, and corticosteroids have considerable effects on T cell trafficking generally, thus likely suppressing GVHD and GVL at the same time (52). Corticosteroids are the first line treatment for both acute and chronic GVHD. However, higher dose and longer duration of corticosteroid therapy is associated with poor outcomes (53–55). Recently, ruxolitinib has been approved for steroid-refractory acute and chronic GVHD, ibrutinib and belumosudil for steroid-refractory chronic GVHD (56). However, it is not clear for how long they should be administered with a fear of losing GVL effects.

## Separation of GVL and GVHD by modulating donor T cell migration

Although host alloantigen is essentially expressed in all tissues, target tissues of acute GVHD are the skin, gut, and liver, suggesting that donor T cells are polarized to traffic to these tissues. Accumulating evidence suggest that intestine is a critical site for alloreactive T cell activation by APCs (23, 57–59). The α4β7 integrin-MAdCAM (mucosal addressin cell adhesion molecule)-1 interactions are essential for donor T cell homing to the gut, and the subsequent development of lethal GVHD (57, 59, 60). A phase III randomized, double-blind, placebo-controlled study (GRAPHITE study) evaluated the efficacy and safety of vedolizumab, a humanized monoclonal antibody directed against α4β7 integrin, which is expressed on T cells and is essential for gastrointestinal (GI) trafficking (61). This study met its primary endpoint by demonstrating a higher lower GI-GvHD-free survival with a comparable safety profile (presented at 2023 Transplantation & Cellular Therapy Meetings; Abstract# LBA2). The impacts on skin and liver GVHD, as well as leukemia relapse, remain to be investigated.

The C–C chemokine receptor 5 (CCR5) interacts with multiple chemokine ligands that mediate the migration and function of T cells and other immune cells to the inflamed tissues (62). CCR5 is critical for donor T cell recruitment to tissues involved in visceral acute GVHD (57, 63–65). In a clinical study of CCR5 antagonist maraviroc involving 38 patients, none of the patients developed acute liver or gut GVHD (66). However, maraviroc failed to reduce GVHD in a randomized phase 2 trial (67).

The CT10 regulator of kinase (Crk) is a crucial adaptor protein for T cell migration (68). Crk deficient T cells failed to traffic GVHD target tissues but efficiently eliminate lymphoma cells in the circulation (69). However, Crk deficient T cells failed to clear the same tumor growing in the skin (69). These results suggest that Crk could be a potential target in controlling GVHD and GVL effects against circulating hematopoietic tumors.

Sphingosine-1-phosphate (S1P) is a metabolite of sphingolipid, a component of bio membrane. S1P interacts with S1P receptor types 1 to 5 (S1PR1-5) (70). S1P modulates cellular proliferation, survival, and migration. Fingolimod (FTY720), a multi-S1PR inhibitor of S1PR1 and S1PR3–5, sequestrates T cells within the secondary lymphoid organs (SLOs) (71, 72). Preclinical studies showed that administration of fingolimod ameliorated GVHD by inhibiting donor T cell infiltration to GVHD target organs and facilitating activation-induced cell death of alloreactive T cells (73, 74). Unfortunately, fingolimod exerts cardiovascular adverse effects that are accelerated in inflammatory milieu in GVHD through its affinity to S1PR3 (75). T cells primarily express S1PR1 (70). Mocravimod (KRP203), a selective agonist of S1PR1, induced apoptosis of donor T cells in the SLOs, suppressed donor T cell migration into the intestine and skin, and ameliorated GVHD (76). Importantly, mocravimod significantly preserved GVL effects compared to cyclosporine (76). In a phase 1 trial, mocravimod reduced circulating lymphocyte numbers, while increased T cell accumulation in the bone marrow (77, 78). These preliminary results suggest that selective inhibition of S1P and S1PR1 interactions inhibits donor T cell migration to the GVHD target tissues, while preserving its migration to the bone marrow. A global phase 3 study evaluating mocravimod in patients with acute myelogenous leukemias (AML) is ongoing.

Ruxolitinib, a JAK1/2 inhibitor, has been approved for the treatment of corticosteroid-refractory acute and chronic GVHD (79, 80). Ruxolitinib suppresses donor T-cell infiltration in the GVHD target organs by reducing CXCR3 expression on donor T cells (81, 82).

Such a strategy of modifying donor T cell migration may eliminate the use of broad immunosuppression, thereby minimizing infectious complications and preserving GVL effect (**Figure 1**).

## Separation of GVL and GVHD through localized GVHD therapy

In mouse models, alloreactive donor T cells migrate into target tissues early after allogeneic HCT. However, late-phase GVHD is maintained within affected tissues locally by tissue-resident TCF1^+^ pTex-like cells without migrating between target organs (83). In patients with multiple sites of GVHD, the dominant T cell receptor repertoires are not consistently observed across tissues within the same patient, raising the possibility that GVHD arose in each local tissue independently (84). Furthermore, patient-derived resident memory T cells persist for many years after HCT in association with the development of skin GVHD (85). These results suggest that tissue-resident memory T cells could be a therapeutic target for tissue-specific GVHD (**Figure 1**). Strategies that prevent T-cell migration into GVHD target organs could also inhibit the development of donor-derived tissue resident memory T cells. In addition, the local therapies also suppress the activities of tissue resident T cells.

**Figure 2:**
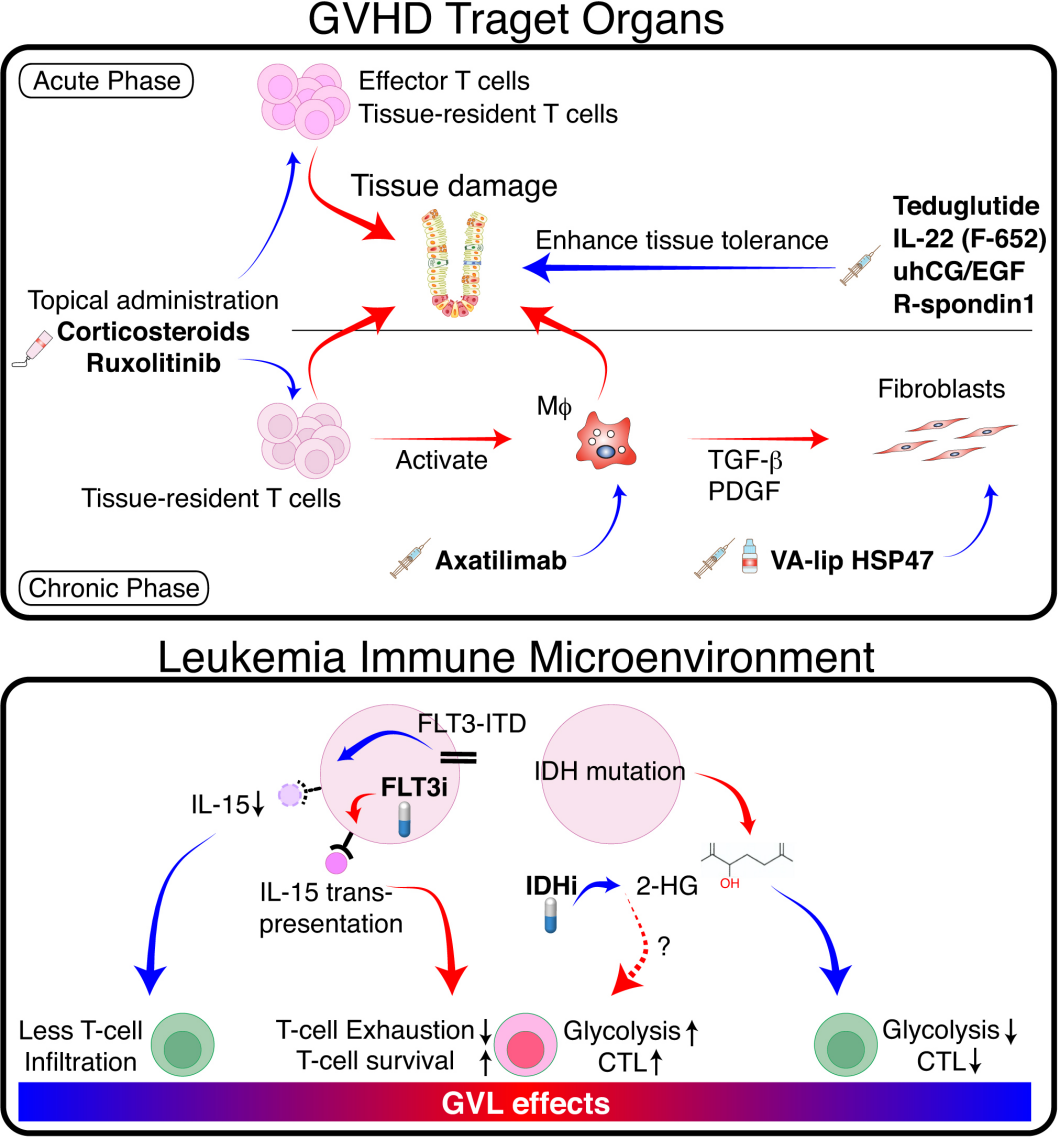
Separation of GVL from GVHD by tissue specific GVHD therapy and modulation of TIME. (Upper panel) In GVHD target organs, donor T cells and innate immune cells such as macrophages (Mϕ) cause tissue damage via production of proinflammatory cytokines and direct cytotoxicity. Mϕ produce pro-fibrotic cytokines such as TGF-β and platelet-derived growth factor (PDGF), which activate fibroblasts and promote pathologic fibrosis and remodeling. Topical immunosuppressants such as corticosteroids and ruxolitinib suppress GVHD locally without impeding GVL. Mϕ-targeting agents such as CSF-1 receptor antibodies, and fibroblast-targeting agents such as VA-lip HSP47 may ameliorate chronic GVHD without affecting GVL effects. Growth factors for tissue stem cells or epithelial cells promote tissue tolerance, thereby suppressing tissue damage and facilitating tissue regeneration. (Lower panel) FLT3-ITD signaling produces immune-cold TIME in FLT3-ITD-expressing AML by inhibiting leukemia production of IL-15. FLT3 inhibitors restore IL-15 production and activate neighboring T cells. Mutated IDH produces 2-HG, which inhibits glycolysis and reduces cytotoxicity in neighboring T cells. IDH inhibitors activate T cells locally by reducing 2-HG and likely promote GVL effects. Red arrows indicate positive regulation, while blue arrows indicate negative regulation.

While topical corticosteroids have been used for treating mild GVHD in the skin and gastrointestinal tract without blunting GVL effects, they are inadequate for treating severe GVHD. Enhancing local GVHD therapy could be the straightforward approach to dissect GVL and GVHD (**Figure 2**). We have recently discovered that topical ruxolitinib effectively improved skin GVHD in mice, while topical corticosteroids exhibited direct toxicity against skin stem cells (86). Long-term use of corticosteroids induces adverse effects on the skin, such as skin atrophy and delayed wound healing (87). In contrast, ruxolitinib protects skin stem cells from GVHD and facilitates wound healing (86). Ruxolitinib cream is now being tested for chronic skin GVHD in a randomized clinical trial.

Fibrosis is an end-stage consequence of chronic inflammation in chronic GVHD. Treatment of fibrotic chronic GVHD with anti-fibrotic agents may not impede GVL (**Figure 2**) (88). A vitamin A-coupled liposome containing siRNA against heat-shock protein 47 (VA-lip HSP47) delivers HSP47 siRNA to pathogenic myofibroblasts in affected organs, such as the skin and salivary glands, and ameliorates fibrosis in mouse chronic GVHD (89). Macrophage-targeting therapy can also improve fibrosis by inhibiting the production of a pro-fibrotic cytokine TGF-β in mice (89, 90). Such approach likely has a minimal impact on GVL effects; axatilimab, an anti-CSF1R monoclonal antibody, is being tested in a clinical trial for chronic GVHD (91). Topical therapy using anti-fibrotic agents is also a promising treatment option. Fibrosis of the lacrimal glands leads to dry eye syndrome in chronic GVHD (92). Ocular instillation of VA-lip HSP47 ameliorates dry eye syndrome in chronic GVHD by targeting myofibroblasts in the lacrimal glands in mice (93).

## Separation of GVL and GVHD by promoting tissue tolerance

Recently, ‘tissue tolerance’ has been proposed as a concept to comprehensively understand the mechanisms enhancing tissue resilience and regeneration during immune reactions (94). The sensitivity of target tissues to GVHD may be modulated by tissue-intrinsic resilience and homeostasis (95, 96). Modulation of GVHD by increasing tissue tolerance would be a promising adjunct therapy without impeding GVL (**Figure 2**). We and others have demonstrated that acute GVHD targets tissue epithelial stem cells in the intestine and skin, leading to prolonged and refractory GVHD (86, 95, 97, 98). Protection and stimulation of tissue stem cells to improve tissue tolerance and repair may represent a novel adjunct strategy for separating GVHD and GVL. For example, since IFN-γ damages tissue stem cells in the intestine and skin, ruxolitinib has the potential to improve GVHD by both suppressing immune reactions and protecting tissue stem cells (86, 99). R-Spondin3 is produced by intestinal stromal cells and lymphatic endothelial cells to maintain intestinal homeostasis by stimulating proliferation and differentiation of LGR5^+^ intestinal stem cells (100–104). Lymphatic endothelial cell injury in GVHD impairs R-Spondin3 production (102). Administration of recombinant R-Spondin1 stimulate growth and differentiation of intestinal stem cells, thus ameliorating experimental GVHD (97, 105). Similarly, administration of glucagon-like peptide-2 (GLP-2), a growth factor for intestinal stem cells produced by enteroendocrine cells in the intestine, ameliorates experimental GVHD (106). Teduglutide, a dipeptidyl peptidase inhibitor 4 (DPP4)-resistant analog of GLP-2, protects intestinal stem cells and enhances the barrier effects of the intestinal mucosa in experimental GVHD (106). IL-22 is produced by type 3 innate lymphoid cells, and its levels are reduced in acute GVHD (98). F562, a fusion protein containing two IL-22 molecules and IgG2-Fc, enhances epithelial regeneration in experimental GVHD and is currently under development in clinical studies (107, 108). Epithelial growth factors could also have beneficial effects on tissue tolerance. Administration of IL-25, a growth factor for goblet cells, mitigates the disruption of mucus layer of the intestine, and ameliorates experimental acute GVHD (109). Urinary-derived human chorionic gonadotropin/epidermal growth factor (uhCG/EGF; Pregnyl) contains abundant EGF, which protects the gut epithelium. A phase 2 trial of Pegnyl showed promising results (110). In conclusion, these therapies targeting tissue tolerance hold promise as treatment strategies for acute GVHD without inducing general immunosuppression and impeding GVL.

## Separation of GVL and GVHD by targeting leukemia microenvironment

Internal tandem duplications (ITD) of the receptor-tyrosine kinase FLT3 gene (FLT3-ITD) are found in 20–25% of AML, providing a persistent growth stimulus. Tumor immune microenvironment (TIME) impacts on the outcome of immune-mediated treatment of various cancers. It has been shown that FLT3-ITD expressing AML has “immune cold” TIME with significantly less T and NK cell infiltration in the bone marrow compared to other types of AML (111). This is possibly due to the inhibition of IL-15 production by FLT3-ITD signaling in AML cells (112). A multi-tyrosine kinase inhibitor sorafenib restores IL-15-production in FLT3-ITD^+^ AML cells by inhibiting FLT3-ITD signaling (**Figure 2**) (112). IL-15 is a homeostatic cytokine for CD8^+^ T cells and plays a critical role in the survival and activation of CD8^+^ T cells. The biologic activities of IL-15 are uniquely mediated by the IL-15-IL15Rα complex produced by non-T cells and “trans-presented” to neighboring CD8^+^ T cells (113). Thus, IL-15 produced by FLT3-ITD^+^ AML cells can activate neighboring CD8^+^ T cells in the bone marrow, thereby promoting GVL effect without significant GVHD induction (112). A subsequent study demonstrated that a selective FLT3 inhibitor gilteritinib also promoted GVL effect without exacerbating GVHD through the similar mechanisms (114). However, in patients with liver involvement of FLT3-ITD^+^ AML, FLT3 inhibitor may exacerbate hepatic GVHD by stimulating neighboring T cells. Another immunosuppressive TIME could be produced by IDH mutation. IDH1-mutation in gliomas suppressed local expansion and cytotoxicity of CD8^+^ T cells by producing oncometabolite D-2-hydroxyglutarate (2-HG) (115). This localized T-cell suppression can be promptly reversed by IDH-inhibition. Although the role of IDH1 mutation on TIME is less clear in AML (116), these results suggest that IDH inhibitors may induce localized GVL effects around AML cells without affecting systemic GVHD.

## Conclusions

Isolating GVL effects from GVHD has been a paramount issue in transplant community. However, redirecting the donor T cell alloreactivity specifically towards leukemia cells, while avoiding exacerbation of GVHD, remains challenging. In this context, the prospect lies in enhancing localized immune suppressive therapies and impeding T cell migration into GVHD target organs, while maintaining T cell migration toward the bone marrow, where niches for leukemic stem cells exist. In addition, improving our understanding of the biology of tissue stem cells in GVHD target tissues will facilitate the development of therapies aimed at promoting tissue tolerance. Counteracting immune evasion of leukemia cells stands as another avenue for enhancing GVL effects. Particularly, specific inhibitors targeting mutated molecules within leukemia may amplifying GVL effects by modulating TIME. Better understanding of the mechanisms of GVHD and GVL is essential to develop strategies for separating GVHD and GVL.

## Author contributions

TT: Writing – original draft. DH: Writing – original draft.
